# The Gender-Related Impact of a Violence Management Training Program on Medical School Students—Preliminary Results

**DOI:** 10.3390/ijerph17197130

**Published:** 2020-09-29

**Authors:** Jakub Lickiewicz, Paweł Jagielski, Patricia Paulsen Hughes, Marta Makara-Studzińska

**Affiliations:** 1 Department of Health Psychology, Institute of Nursing and Midwifery, Faculty of Health Sciences, Jagiellonian University Medical College, 31-501 Krakow, Poland; marta.makara-studzinska@uj.edu.pl; 2 Department of Nutrition and Drug Research, Jagiellonian University Medical College, 31-501 Krakow, Poland; paweljan.jagielski@uj.edu.pl; 3 College of Education, Health, and Aviation, Oklahoma State University, Stillwater, OK 74078, USA; trish.hughes@okstate.edu

**Keywords:** violence, aggression, intervention, violence management training, student

## Abstract

Phenomenon: Patient aggression directed toward medical personnel, including medical school students during their internships, is an increasingly important issue. To minimize this phenomenon, violence management training programs were carried out. Approach: To assess the efficacy of a violence management training program among medical school students and evaluate changes in the perception of aggressive behavior in relation to the participants’ sense of self-efficacy and self-confidence by sex. A quasi-experimental examination of medical school students was performed before and after completion of a training program. Two hundred seventy-six students, including students of medicine, nursing, emergency medical services, and physiotherapy, participated in the study. Three standardized questionnaires were used: The Perception of Aggression Scale (POAS), the Hope for Success Questionnaire (HSQ), and the General Self -Efficacy Scale (GSES). Findings: The training program had a positive impact on the sense of self-efficacy in both men and women. However, the perception of aggressive behavior changed only in women and the impact of such intervention was higher for women. Further studies should look at the long-term outcomes.

## 1. Introduction

Patient aggression is one of the major challenges that healthcare staff face on a daily basis that has negative consequences for staff and patients alike [1,2]. Research shows that healthcare staff members perceive aggression as part of their work [3]. The perception of aggression depends, among other factors, on a sense of insecurity, which in turn seems to be related to the frequency of aggressive behavior [4]. Verbal aggression consisting of screaming and threats occurs most frequently. Contact with aggressive patients and their families tends to generate negative emotions among medical staff such as anger, frustration, a sense of helplessness, self-accusation, and, consequently, a negative attitude toward aggressive patients [5,6]. The most frequent results are work absenteeism, changing workplaces, burnout, or even leaving health care altogether [7]. Such behavior also affects medical school students, particularly women [8], who most often perceive aggressive behavior as violence. Perception of such behavior is largely sex-dependent. Female students perceive aggressive behavior more negatively than their male counterparts [9,10].

To counteract the negative impact of patient aggression, some actions can be taken that focus on introducing changes to the legal system and monitoring the incidence of aggressive behavior. One of the preventive methods is to educate medical staff, training them specifically to deal with patient aggression [11]. These are known as aggression management programs (AMPs) [12]. However, so far, there has been no universal methodology for such training; the focus has shifted from physical defense to preventive techniques. Presently, the focus is on understanding the mechanism of aggression that may govern aggressive behavior. Such an approach involves a calm demeanor toward an aggressive patient or client, which in turn facilitates dealing with challenging behavior as well as preventing it. The training helps staff members understand their own perception of violence and potential interventions in hypothetical situations [13]. The training also delivers practical advice for dealing with aggressive patients. However, the ultimate goal is not to deliver skills to deal with challenging behavior, but rather to develop knowledge and skills to prevent such behavior from occurring in the first place. The main focus is to teach proactive behavior.

Presently, the syllabi of the training programs cover work environment and management principles, theories of and motivations for aggression, estimating risk, communication and conflict de-escalation principles, physical techniques for intervention and coercive measures, ethical and legal issues, and the steps to be taken after aggression occurs [14].

Therapeutic Management of Aggression (TERMA) is one of the training programs used to prevent and manage aggression. It was developed in the Department of Forensic Psychiatry at Haukeland University Hospital in Bergen, Norway. The curriculum includes: the theory of aggression, emotional and physical reasons for aggression, identification of one’s thought patterns when dealing with aggression, and risk factors for violent behavior in hospitals. Additionally, participants become familiar with ethical and legal issues related to aggressive behavior as well as the role of personnel group work in dealing with patient aggression. This course also offers physical self-defense training as well as periodical revision of course content. The principles of TERMA have also been applied outside of Norway- in Poland [15] and Sweden, where they are known as the Bergen model [16]. The theoretical framework of TERMA is based mostly on the City model by Leo Bowers. It suggests that effective aggression prevention has three elements: positive appreciation of patients (moral perception and compassion), emotional regulation (suppression and emotional equilibrium), and effective structure (routine direction and conduct). To meet these goals, medical personnel need to understand the importance of teamwork, self- management, and organizational support [16].

The question often posed while discussing training concerns their effectiveness. Training focused solely on elements of physical self-defense appears to be ineffective. Research indicates an inability to successfully defend against aggressive behavior in 40% to 80% of participants [17,18]. Nevertheless, some attempts to adapt self-defense programs to the hospital environment have appeared, which have resulted in a sense of security among the staff [19]. This, in turn, indicates that self-defense training leads to an increasing sense of security among the staff. However, evaluation of the actual effectiveness of such programs requires further research.

The efficacy of AMPs is clear, bearing in mind that the scope of such research is not limited only to elements of physical self-defense. Research indicates a positive impact of AMPs on job satisfaction [20] and the quality of patient care [21]; confidence, anxiety, and a sense of self-trust [19]; attitudes toward and the perception of aggression; and the level of efficacy when managing aggressive patients [22,23]. Increased confidence when dealing with aggressive behavior [24,25] and increased ability to cope with stress is emphasized. A subjectively assessed development of useful skills for dealing with aggression has also been revealed [26]. Other research indicates fewer aggressive incidents following an aggression prevention program, although the results were not statistically significant [5]. Aggression prevention programs have also been offered to medical students, which has resulted in the development of skills to de-escalate aggressive patient behavior [27]. Similar results have been reported in anger management training, which has resulted in the development of skills to control negative emotions [28].

Some researchers question the efficacy of AMPs. Heckemann [29] saw unchanged attitudes despite noticeable skill and knowledge development regarding aggressive behavior in patients. However, the number of aggressive incidents in hospital wards remained unchanged, with researchers attributing the lack of change to the nurses participating in the training not sharing their knowledge with other staff members. Thus, Heckemann and colleagues concluded that introducing organizational changes was more important than training staff.

The effectiveness of AMPs should be evaluated in relation to their objectives, which are mostly to deal with aggressive patients and teach skills useful for preventing aggressive behavior [30]. AMPs as a method enhance changes in attitudes, which in turn lead to changes in behavior. Consequently, in order to reduce the number of aggressive incidences, it is necessary to impact service provision and organizational policies.

To date, researchers have not considered the impact of aggression prevention trainings on self-efficacy or self-esteem, but rather have focused mainly on the sense of efficacy toward aggressive patients. When analyzing AMP efficacy, numerous facets should be considered and not limited to the number of aggressive incidents.

Training should offer knowledge on preventive techniques and potential reactions but also awareness of which options are most appropriate to apply under different circumstances. Thus, training focuses mainly on knowledge. However, it may also impact attitudes toward and the perception of aggressive behavior. Therefore, it should be assumed that aggression prevention trainings have a positive impact on medical staff and medical students by offering not only a stronger sense of self-confidence in a confrontational situation with a patient, but also by influencing other psychological traits.

The question arises whether the effectiveness of AMPs is sex-related or if the perception of aggression is sex-dependent. The purpose of the current study was to assess sex-based efficacy of a violence prevention program, as well as to evaluate changes in the perception of aggressive behavior in relation to self-efficacy and self-confidence.

There is a lack of theories explaining AMP training in relation to the different sexes. Gender is important in women’s self-defense. The feminist approach provides better insight into gender differences in relation to aggression. According to this concept, women’s and men’s perceptions of aggression are closely connected to cultural socialization and expectations of masculine and feminine sex roles [31]. Connell and Messerschmidt suggested that the concept of gender hierarchy, masculinity, and male hegemony should be perceived on a local, regional, and global level [32]. Poland is a rather paternalistic country, and as a consequence, cultural forces socialize men as masculine and women as feminine individuals [33]. Unfortunately, no individual is immune to these long-standing forces in society and the result is gender inequality and the oppression of women. According to Burgess-Proctor (2006), power, privilege, and oppression are multiplicative and intersect according to many social characteristics, including gender, nationality, and age [34]. As a result, women’s self-efficacy and self-confidence in relation to violence and aggression differ from those of men. According to Lazarus, there is a gap between gender and crime that also applies to medical personnel [31]. Female medical personnel can expect certain types of aggression (e.g., sexual) in addition to what men experience [8,10]. For this reason, there is a need to better understand gender- related issues in AMP training.

## 2. Materials and Methods

The study population included 276 students from Jagiellonian University Medical College. Out of 316 questionnaires, 40 were rejected, i.e., 7.9%, mainly due to being incomplete. The study procedure was approved by the Bioethical Committee at Jagiellonian University Medical College.

The training program was run by one person to avoid potential interference from any extraneous variables connected with the trainer’s personality or training style. The training used the TERMA methodology and curriculum. It consisted of 30 training sessions divided into 5 themes. The participants were acquainted with psychological mechanisms of aggressive behavior, aggression de-escalation methods, legal regulations and breakaway techniques. TERMA includes rules for applying coercive measures such as restraints. Due to the composition of the group (students, rather than professional medical personnel), practical training in the use of coercive means was excluded, thus limiting the topic to psychological and legal aspects. Psychological issues took 20 h, and the remaining time (10 h) was devoted to physical techniques. The training consisted of lectures, seminars, case studies, and role-playing.

The data acquisition procedure was divided into two phases. Initially, the participants completed the Perception of Aggression Scale (POAS), the General Self-Efficacy Scale (GSES), and the Hope for Success Questionnaire (HSQ, a pre-intervention paper questionnaire) [35,36,37,38]. The participants copied the unique number on each questionnaire onto a blank sheet. Then they marked the sheet with a symbol of their choice, which later allowed for the identification of each person’s data. Within two weeks of completing the training, the participants completed the same questionnaires again. Subsequent questionnaires were coded to enable the responses given before and after the intervention to be matched, which ensured the participants’ anonymity and met the criteria for using statistical tests with dependent samples.

As the variables under consideration did not have normal distribution, the Shapiro–Wilk test of normality was used. To examine the homogeneity of the variance between groups, the ANOVA Kruskal–Wallis test was used. The intervention effectiveness was measured with the Wilcoxon signed-rank test. The significance of the difference in participants’ gender and faculties was evaluated with the Mann–Whitney U test and the ANOVA Kruskal–Wallis test. In all calculations, IBM SPSS Statistics 25 for Windows software (IBM Corp, Armonk, NY, USA) was used, and statistical significance was set at *p* ≤ 0.05.

## 3. Results

The 276 participants in this study were students at Jagiellonian University Medical College: 170 (61.6%) in medicine, 57 (20.7%) in nursing and midwifery, 26 (9.4%) in physiotherapy, and 23 (8.3%) in emergency medical services. As no statistically relevant demographic differences were reported among the participants with regard to academic discipline, the group was treated as homogenous. The group consisted of 206 female and 70 male students (74.6% and 25.4%, respectively), and the average age was 22.1 ± 4.62 years. A comparison made between male and female groups indicated statistically relevant differences in relation to the variables tested. Table 1 shows the differences between the two comparison groups from before the training and Table 2 shows results from after the training.

The sex intergroup differences observed before the intervention showed that women perceived aggression as a kind of dysfunctional behavior, whereas men saw it as protective behavior aimed at defense of territory. After the intervention, no statistically relevant differences appeared. Women still perceived aggression as protective behavior to a lesser extent than men, but the sex difference was no longer statistically significant.

Subsequently, the impact of the intervention on psychological traits and attitudes toward aggressive behavior was analyzed. Table 3 shows the results for women and Table 4 shows the results for men.

In the group of women, the increases in their sense of self-efficacy and self-confidence were significant. A change occurred in the perception of aggression, which was considered less dysfunctional behavior, but also less frequently considered functional behavior. After the training participants treated aggression as self-protection.

In the group of male participants, the training had a positive impact on self-efficacy and self-confidence. No statistically relevant differences were shown in aggression perception.

## 4. Discussion

We explored sex differences in the perception of aggressive behavior. When compared to women, men initially perceived aggression as a less destructive and more protective kind of behavior. These results are consistent with the earlier findings of Bilgin and Keser [10] on culturally determined gender roles of the man showing and managing aggression, and the woman as falling victim to aggression. The differences should be analyzed in a broader context, including the concept of masculinity/femininity and gender inequality [34] (Burgess-Proctor, 2006). The study showed that women tended to consider aggression to be destructive behavior more often than men, which may be due to their fear of violent behavior and lack of confidence in dealing with it [39]. Whereas men tend to justify aggressive behavior as stereotypically masculine, women see it as menacing and impossible to justify. These results may be closely connected to feminism and masculinity and its relationship to women’s perception of aggression [34]. From a feminist perspective, women tend to perceive themselves as ineffective at dealing with aggression because of social stigma and stereotypes [32].

The differences between male and female participants in perception of aggression diminished as a result of the training. As a consequence of the intervention, men regarded violent behavior as more destructive and less protective than before the training. Male participants did not perceive aggression differently; more changes were observed in the group of female participants.

The training proved to be effective for helping both groups change their assessments of their capabilities and perception of aggression. The training impacted women more, increasing their self-confidence, self-efficacy, and hope for success. Simultaneously, it led to a more negative perception of aggressive behavior and women interpreted aggression more positively. After the training, the female participants perceived aggressive behavior as more functional than before.

The question arises about the intended result of the intervention and which effect is more appropriate: leading men to understand the negative aspects of aggression or helping women realize its multifaceted nature. The effect achieved with the latter group consisted of gaining a more negative perception of aggressive behavior concurrent with acknowledging its more complex function, e.g., self-protection or communication. No such result was observed in the men, who admitted an increase in self-confidence and self-efficacy but showed no change in their approach to aggressive patients. This might be connected with masculinity and their confidence in dealing with violence and aggression.

We propose that the training module that dealt with managing physical aggression may have positively influenced the men. As physical fighting is stereotypically masculine, this may have increased men’s self-efficacy in a variety of situations despite being covered only briefly. Earlier studies also reported the impact of such training on the sense of self-efficacy in dealing with difficult patients [27]. From the feminist perspective, women find it harder to change their attitudes and sense of self-efficacy because of deeply rooted feminine stereotypes and the social roles they fulfill [31].

The questionnaires used in this study were based on the social cognitive theory proposed by Albert Bandura. For this reason, the effectiveness of the training concerns the sense of one’s efficacy in difficult situations rather than the accompanying emotions [40]. The aim of the training was to teach participants how to deal with aggressive behavior, which affected their cognitive schemata. Further research is needed to establish the impact of the training upon the participants’ emotions.

The participants’ ability to react to aggression with compassion and empathy is an important element of education [30]. Further exploration is necessary to establish to what extent teaching suitable aggression de-escalation methods (prevention), compared to teaching intervention methods (self-defense), affects the sense of self-efficacy. However, only knowing physical self-defense techniques may increase the sense of self-efficacy, which is misleading because it does not provide real safety.

The study had some limitations. The research focused on participants and changes in their attitudes. It would be beneficial to add a comparison group without training. The information about participants’ expectations about the training would help in understanding the motives behind the decision to attend AMP. It is possible that sex also impacted participants’ expectations.

The training affected the perception of aggression in the female group only. After the intervention, female participants saw aggressive behavior as defense of territory and behavior that served a particular purpose. At the same time, however, they perceived it as less acceptable. The changing perception may have resulted from a better understanding of the motivation behind and causes of aggression. No such effect was observed in the male group, which may be due to their lesser insight or weaker attempts to understand aggression mechanisms. This finding suggests that in aggressive-behavior trainings, methodology should be reviewed and participants divided by sex. As mentioned before, the concept of masculinity and hegemony depends on local and global factors. It would be beneficial to measure the expectation and effectiveness of AMP training in different cultural circles.

As Hollander stated, “feminist or empowerment self-defense is the only type of women’s self-defense training, which has received a rigorous evaluation and as a consequence, a more widely endorsed self-defense” [41]. This would necessitate creating separate programs focused on various aspects of aggression prevention, especially in AMP trainings for medical personnel. Whether training should be provided by sex-matched instructors also remains to be determined.

## 5. Conclusions

The results concerning psychological features appear to be the measure of self-efficacy in managing patient aggression. Consequently, the training affected self-efficacy and trust in one’s capabilities. Further studies should look at long-term outcomes. The material covered during the course should also be reviewed after the course.

The intervention proved effective, especially for female participants. This may be the result of challenging stereotypes and broadening female participants’ knowledge of how to manage patient aggression. The perception of aggression and the ability to deal with it differs because of gender inequality. AMP instructors should consider this aspect when creating the training curriculum. The effort of the women who attend the training differed from that of the men. Women have to deal with their own beliefs about gender equality. This kind of training plays an important role in medical staff education and should not be marginalized.

## Figures and Tables

**Table 1:** Differences between male and female participants before the intervention (n = 276).

	Female		Male		Test Value (*p*)
	X	Me ± Q	X	Me ± Q	
General Self-Efficacy Scale (GSES)	28.52	29.0 ± 2.0	29.13	29.0 ± 1.5	0.14
Hope for Success Questionnaire (HSQ) overall score	47.88	48.0 ± 3.5	48.44	49.0 ± 4.0	0.58
HSQ FS	24.68	25.0 ± 1.5	25.43	26.0 ± 1.5	0.17
HSQ WP	23.20	24.0 ± 1.5	23.02	23.0 ± 2.0	0.77
POAS Dysfunctional	11.47	11.0 ± 1.5	12.73	12.0 ± 2.5	**0.02**
POAS Functional	16.50	17.0 ± 0.5	16.16	16.0 ± 1.0	0.36
POAS Protective	5.62	5.0 ± 1.0	4.71	4.0 ± 1.0	**<0.01**

N = number, X = average, Me = median, Q = quartile deviation, *p*-value = Mann–Whitney U test. Bold = statistically significant results (<0.05).

**Table 2:** Differences between male and female participants after the intervention (n = 276).

	Female		Male		Test Value (*p*)
	X	Me ± Q	X	Me ± Q	
GSES	29.79	29.5 ± 2.0	29.82	30.0 ± 2.0	0.63
HSQ overall score	50.73	51.0 ± 4.5	50.59	50.0 ± 3.5	0.79
HSQ FS	25.93	26.0 ± 2.5	26.2	27.0 ± 1.0	0.66
HSQ WP	24.8	25.0 ± 3.0	24.39	24.0 ± 2.0	0.61
POAS Dysfunctional	11.04	10.0 ± 2.5	11.96	11.0 ± 3.5	0.15
POAS Functional	15.92	16.0 ± 2.5	15.66	16.0 ± 2.0	0.17
POAS Protective	5.29	5.0 ± 1.5	4.69	4.0 ± 1.0	**0.04**

N = number, X = average, Me = median, Q = quartile deviation, *p*-value = Mann–Whitney U test. Bold = statistically significant results (<0.05).

**Table 3:** Impact of the intervention on personality traits and aggression perception in female participants (n = 206).

	Before		After		Difference		Test Value (*p*)
	X	Me ± Q	X	Me ± Q	X	Me ± Q	
GSES	28.52	29.0 ± 1.5	29.79	29.5 ± 2.0	1.26	1.0 ± 2.0	**<0.01**
HSQ overall score	47.88	48.0 ± 4.5	50.73	51.0 ± 4.5	2.89	3.0 ± 3.0	**<0.01**
HSQ FS	24.68	25.0 ± 2.0	25.93	26.0 ± 2.5	1.27	1.0 ± 2.0	**<0.01**
HSQ WP	23.20	24.0 ± 2.5	24.8	25.0 ± 3.0	1.62	1.0 ± 1.5	**<0.01**
POAS Dysfunctional	11.47	11.0 ± 2.5	11.04	10.0 ± 2.5	−0.42	−1.0 ± 2.5	**0.02**
POAS Functional	16.50	17.0 ± 2.0	15.92	16.0 ± 2.5	−0.58	0.0 ± 1.5	**0.04**
POAS Protective	5.62	5.0 ± 1.5	5.29	5.0 ± 1.5	−0.33	0.0 ± 1.5	**0.03**

N = number, Me = median, Q = quartile deviation, *p*-value = Wilcoxon signed-rank test value. Bold = statistically significant results (<0.05).

**Table 4:** Impact of the intervention on personality traits and aggression perception in male participants (n = 70).

	Before		After		Difference		Test Value (*p*)
	X	Me ± Q	X	Me ± Q	X	Me ± Q	
GSES	29.13	29.0 ± 2.0	29.82	30.0 ± 2.0	0.58	0.0 ± 1.0	**0.01**
HSQ overall score	48.44	49.0 ± 3.0	50.59	50.0 ± 3.5	2.15	2.0 ± 2.5	**<0.01**
HSQ FS	25.43	26.0 ± 2.0	26.20	27.0 ± 1.0	0.78	1.0 ± 1.5	**0.03**
HSQ WP	23.02	23.0 ± 2.5	24.34	24.0 ± 2.0	1.37	1.0 ± 2.0	**<0.01**
POAS Dysfunctional	12.73	12.0 ± 2.5	11.96	11.0 ± 3.5	−0.77	0.0 ± 2.0	0.17
POAS Functional	16.16	16.0 ± 2.0	15.66	16.0 ± 2.0	−0.50	0.0 ± 1.5	0.15
POAS Protective	4.71	4.0 ± 1.0	4.69	4.0 ± 1.0	−0.03	0.0 ± 1.0	0.86

N = number, Me = median, Q = quartile deviation, *p*-value = Wilcoxon signed-rank test value. Bold = statistically significant results (<0.05).
